# A Novel Method of Failure Sample Selection for Electrical Systems Using Ant Colony Optimization

**DOI:** 10.1155/2016/4238734

**Published:** 2016-09-22

**Authors:** Jian Xiong, Shulin Tian, Chenglin Yang, Cheng Liu

**Affiliations:** ^1^School of Automation Engineering, University of Electronic Science and Technology of China, Chengdu 611731, China; ^2^Department of Communication Engineering, Chengdu Technological University, Chengdu 611731, China

## Abstract

The influence of failure propagation is ignored in failure sample selection based on traditional testability demonstration experiment method. Traditional failure sample selection generally causes the omission of some failures during the selection and this phenomenon could lead to some fearful risks of usage because these failures will lead to serious propagation failures. This paper proposes a new failure sample selection method to solve the problem. First, the method uses a directed graph and ant colony optimization (ACO) to obtain a subsequent failure propagation set (SFPS) based on failure propagation model and then we propose a new failure sample selection method on the basis of the number of SFPS. Compared with traditional sampling plan, this method is able to improve the coverage of testing failure samples, increase the capacity of diagnosis, and decrease the risk of using.

## 1. Introduction

In the process of industrial manufacturing for electrical systems and equipment, testability plays a crucial role in the reliability improvement for large scale electrical equipment [1]. As we know, having good testability of systems and equipment can detect and isolate failures quickly, reduce maintenance time, and increase the availability of the system. Thereby, testability is paid more attentions by many researchers.

Testability refers to testing the abilities of failure diagnosis, fault prognosis, and fault isolation. Numerous models and methodologies have been developed to diagnose, prognose, and prevent failures or faults. In 1983, Huang et al. introduced a new diagnostic concept of K-node fault diagnosis [2]. They pointed out that testability is only relying on the structure of circuits with nothing to do with the value of elements. In [3], Maeda et al. discussed factors of testability and distinguishability for nonlinear systems according to analysis and graph theory. Yang et al. [4] proposed the slope fault mode on the complex field; the method is available for the diagnosis of linear or nonlinear analog circuits. In [5], a new fault diagnostic method under tolerance condition is proposed by using fuzzy math means to detect faults. In order to detect and isolate faulty components and to predict the remaining useful performance of analog circuits, Vasan et al. [6] proposed using a kernel method and a particle filtering method for diagnosis and prognosis, respectively.

One significant stage of design for testability is the testability demonstration experiment. It is to test the ability of failure detection and isolation through injecting some failures [1]. As injected testing failure samples, these failures samples are randomly selected or the selection depends on the biggest probability of failure in traditional methods. However, in accordance with the traditional testability demonstration experiment method failure propagation modes in systems are ignored, which commonly leads to serious fault omission in failure sample selection. In other words, if propagation failures are caused by some failures which have very low failure rate, it means that these failures could not be selected in the traditional testability demonstration experiment because of their lower failure rate. When their failures occur in a system they will spread to other components and cause huge faults. The phenomenon could be a serious problem. That is, if we do not consider propagation failures, these low failure rate faults which cause propagation faults could be missed. This means that established test failure set is not complete and is not able to detect and isolate failures correctly. To solve the problem, numerous related researches have been developed [7–9]. Reference [7] proposed an approach to analyze failure propagation of aircraft engine systems with small world net theory. Li et al. utilized fuzzy probability Petri net model to describe fault propagation and then the method of sample selection based on propagation intensity was introduced [8]. This method can afford better fault coverage rate.

Our work is to employ failure propagation probability to deduce the intensity of failure propagation and then optimize maximum probability failure propagation path using ant colony optimization (ACO) according to the intensity of the failure propagation. Finally, subsequent failure propagation sets (SFPS) are built and a new failure sample selection plan is proposed. The proposed method can effectively reduce the risk of omission of propagation failures, and it increases the accuracy of failure diagnosis.

The materials in this work are arranged as follows. In Section 2, a brief introduction to the principle of failure propagation modeling and ant colony optimization is introduced. Moreover, a new failure sample selection optimization method is presented. In Section 3, a case study is used to verify the failure diagnostic effect of our method through comparing the traditional failure sample selection. Finally, brief conclusions are presented in Section 4.

## 2. Methodology

**Figure 1:**
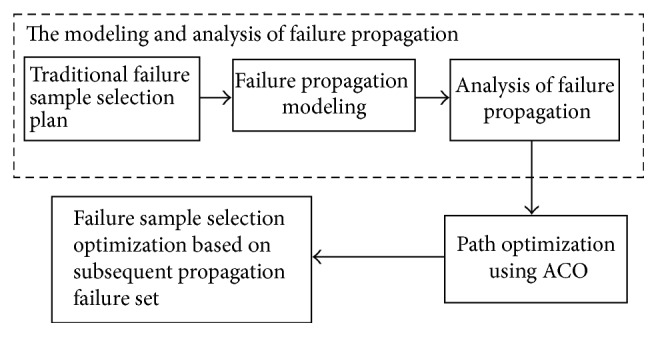
Block diagram of the procedure of optimization.

The proposed method involves three major stages: (1) failure sample selection plan design and analysis on the basis of failure propagation model; (2) path optimization with ACO; and (3) failure sample selection optimization based on subsequent propagation failure set. The block diagram of the procedure of optimization for electrical systems is shown in Figure 1. Once a sampling plan is confirmed, failure samples will be assigned to different modules. In order to detect and identify failures correctly and avoid the omission of propagation failures, failure propagation should be taken into account. Through adopting ant colony optimization algorithm, the maximum probability propagation path is searched. Then a new failure sample selection is proposed according to the intensity of edge in this path. In the section, fundamentals of the procedure of testability demonstration experiment and ACO are provided as below at first.

### 2.1. The Modeling and Analysis of Failure Propagation

#### 2.1.1. Stage 1: The Building of Failure Sample Selection Plan

**Figure 2:**
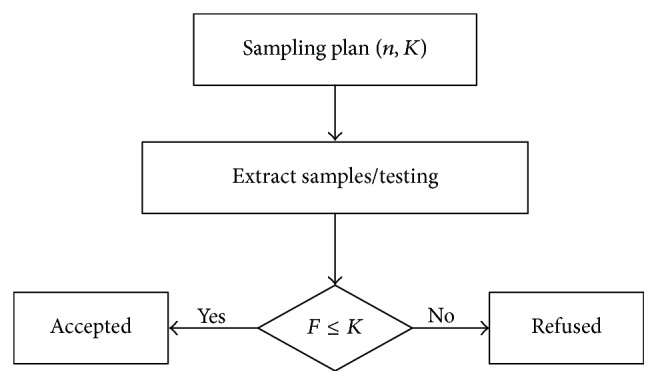
The failure sample selection plan.

Before testability demonstration experiment, we need to extract enough failure samples by using random sampling. Suppose, in a testability demonstration experiment, *n* samples are selected for independence test and *F* tests result in failure. A positive integer *K* is regulated as a threshold value; if *F* ≤ *K*, the experiment is considered up to standard. Otherwise, it is considered unqualified.  Figure 2 shows the process of traditional failure sample selection plan. Thereby, the primary aim of the scheme is to determine the value of *n* and *K*.

For testability demonstration experiment, we assume that it meets the requirements of a binomial distribution. Assume that the probability of success of each test is *q*, after *n* independent tests. The probability of *F* failures can be expressed as

(1) $$\begin{array}{c} p\left(\;q;n,F\;\right)=C_{n}^{F}{\left(\;1-q\;\right)}^{F}q^{n-F}, \end{array}$$

where *C*_*n*_^*F*^ is the combinatorial number. It represents the number of all combinations where each combination is an unordered collection of *F* distinct elements. And these *F* distinct elements are taken from a giving set consisting *n* elements. To our knowledge, for a successful testability demonstration experiment, the number of failure tests should be less than or equal to the threshold value *K*. Therefore, the probability of failure for a successful testability demonstration experiment is equal to the sum of probabilities of its failure tests. The following expression is given by

(2) $$\begin{array}{c} L\left(\;q\;\right)=\overset{K}{\underset{F=0}{\sum }}p\left(\;q;n,F\;\right). \end{array}$$

Through consultation between suppliers and customers, the design value *q*_0_ of fault detection rate (FDR) is determined. The design value is the probability of success for one test. FDR's minimum acceptable value is *q*_1_. When *q*_0_ ≥ *q*_1_, we consider the test has reached the design standard. The suppliers' risk is *α*, which denotes the minimum accepted probability of success for an experiment by suppliers. The risk of using is *β* which is the maximum probability of failure for the experiment. Under these conditions, we can use formula (3) to determine the values of *n* and *K*.

(3) Lq1=∑F=0KCnF1−q1Fq1n−F≤β,1−Lq0=1−∑F=0KCnF1−q0Fq0n−F≥α.

When the plan (*n*, *K*) is confirmed, *n* samples will be assigned to different modules in the system according to layered design pattern and proportions. Then, an injected failure set is built through extracting failure modes in each module. As we all know, we should acquire only *n* failure modes, and the number of these failure modes is less than the total number of failure modes in the systems. In order to guarantee that the injected failure set has bigger failure coverage, a hierarchical distribution of failure sample size is used. Its formula is shown in

(4) ni=n∗Wi,Wi=QiλiTi∑iQiλiTi,λi=1MTBFi,

where *n*_*i*_ denotes the number of assigned samples for module *i*, *W*_*i*_ is the assignment weight of the *i*th module, *Q*_*i*_ is the number of failure modes of the *i*th module and it indicates the complexity of equipment, *T*_*i*_ is the operation time coefficient of the *i*th module and it is equal to the ratio of the operation time and work life, and (MTBF)_*i*_ represents mean time between failures in module *i*. Thus, *λ*_*i*_ is the failure rate in the *i*th module which is expressed in failures per unit of time.

#### 2.1.2. Stage 2: Failure Propagation Modeling

**Figure 3:**
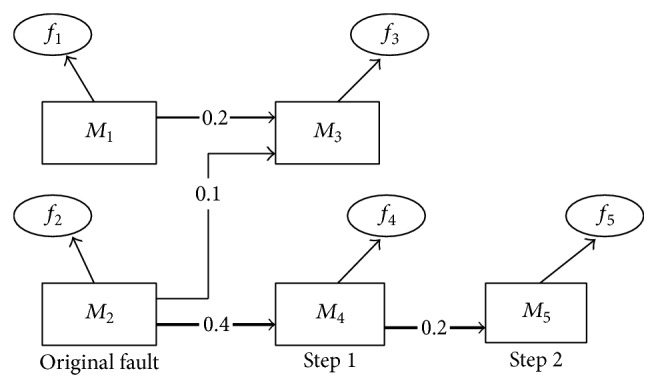
An example of DG.

In this section, a failure propagation model will be built based on propagation probability with the use of directed graph (DG) of failure propagation and adjacency matrix. In graph theory, DG is a graph, which is a set of nodes connected by directed edges. It can be used to describe the relationship of failure propagation among components of electrical system with nodes and directed edges. In formal terms, directed graph is represented with a function DG = {*M*, *F*, *E*} as shown in Figure 3. In the diagram, *M* indicates nodes (components) set; *F* expresses a failure set which includes 5 failure modes such as *f*_1_, *f*_2_,…, *f*_5_; *E* is a set of directed edges which can clearly describe the link and the relationship between any two circuit components or modules with the capacity or intensity of the failure propagation.

These intensities of the failure propagation and relationship between nodes (components) may be heterogeneous. Assuming the system has *N* nodes, we introduce *N* × *N* adjacency matrix *A* = [*a*_*ij*_] to describe the link relationship between components with all zeros on the main diagonal and off-diagonal elements. It is given as follows:
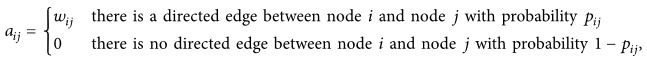
(5)where *w*_*ij*_ is the directed weight between node *i* and node *j* with probability *p*_*ij*_ ∈ [0,1], *w*_*ij*_ ≠ 0 for *i* ≠ *j*,  *w*_*ij*_ = 0 for *i* = *j*, and *i*, *j* ∈ {1, 2,…, *N*}.

The existence of an edge from node *i* to node *j* is determined by the probability *P*_*ij*_ which is independent of other edges. The probability is

(6) $$\begin{array}{c} P_{ij}=\overset{n}{\underset{k=1}{\sum }}u_{k}\left(\;x\;\right)P_{k},\;i,j\in \left\{1,2,\ldots ,N\right\}, \end{array}$$

where *u*_*k*_(*x*) is the membership degree of ambiguity set of failure states, *x* indicates various failure's symptom signals, and *P*_*k*_ represents the probability of the *k*th *x*. The probabilities *p*_*ij*_ are collected in the probability matrix *P* = [*p*_*ij*_].

#### 2.1.3. Stage 3: Analysis of Failure Propagation

When a failure occurs in a certain node of circuit system, the failure spreads to its connected neighbor nodes and could lead to these neighbor node failures. As the directed link weight between nodes, intensity of failure spread indicates the fact that the greater the intensity an edge has, the bigger the possibility that failure propagation happens in the edge. It means that the failure propagation may lead to bigger possibility of cascading failures to its connected neighbor nodes with bigger intensity of edge.

In order to describe the intensity of failure spread, the formula of the intensity is given as follows:

(7) $$\begin{array}{c} I_{ij}^{K}=c_{s}\left[\;w_{p}p_{ij}^{K}+\frac{w_{d}d_{j}^{K}}{\sum_{j\in F_{K}}d_{j}^{K}}\;\right];\;i\in F_{K-1}, \end{array}$$

where *c*_*s*_ is crossing-clustering coefficient. *w*_*p*_ is the weight of failure propagation probability, *w*_*d*_ is the weight of node degree, *p*_*ij*_^*K*^ is the propagation probability from node *i* to node *j* in the *k*th propagation step, *F*_*K*_ represents subsequent node set after *K* propagation steps, and *d*_*j*_^*K*^ indicates the node degree of node *j* in *F*_*K*_. Node degree is the number of edges associated with a node.

In order to easily compare intensities between each other and also to simplify calculations, the *Z*-score of *I*_*ij*_^*K*^ is the most suitable method to compare these intensities in our work, because *Z*-score indicates a datum above or below the mean with signed number. It is defined as

(8) Z=IijK−EIijKσ,

(9) σ=Var⁡IijK,

where *E*[*I*_*ij*_^*K*^] is the expected value of *I*_*ij*_^*K*^ and *σ* is the standard deviation of the population of *I*_*ij*_^*K*^.

For instance, we have known intensities of edges of Figure 3. Their *Z*-scores are calculated by making use of (8) and (9) as shown in Table 1. We take *M*_2_ as an example; according to the structure of the DG, we can see that there are two propagation edges from *M*_2_—namely, edge (*M*_2_, *M*_4_) and edge (*M*_2_, *M*_3_). It is clear that the intensity (1.3908) of the edge (*M*_2_, *M*_4_) is greater than the intensity (−0.9934) of the edge (*M*_2_, *M*_3_). As a result, it is easy to determine that failure *f*_2_ leads to a failure propagation on the edge (*M*_2_, *M*_4_) with greater possibility than on the edge (*M*_2_, *M*_3_).

According to the above analysis, failure propagation happens on the path with the greatest intensity the failure propagation has. As shown in Figure 3, the bold line is *f*_2_'s propagation path with the maximum intensity.

### 2.2. Path Optimization with ACO

In general, the structure of Very Large Scale Integration (VLSI) is very complex and hard to analyze failure propagation through manual work. Hence, intelligent algorithms are used. In order to obtain the maximum probability failure propagation path, the ACO is adopted in the paper.

The algorithm was proposed by M. Dorigo in his doctoral thesis in 1991 and it was aimed at solving the travelling salesman problem based on the action of ants, in which the goal was to find the shortest round-trip to link a series of cities [10]. More details about this technique can be found in [10]. The ACO has strong robustness and it is suitable for parallel implementations [11]. Therefore, we use the ACO to search the maximum probability failure propagation path.

The mathematical model of the maximum probability failure propagation path in circuits can be represented as follows:

(10) max⁡ ∑KdK;K=1,2,…,ns.t. ∏KpijK≤10−8;i∈FK−1;  j∈FK.

At a given time *t*, ants make use of pheromone which is deposited between nodes to search subsequent path from node *i*. For ant *K*, the probability of selected next path is

(11) $$\begin{array}{c} P_{ij}^{K}=\left\{\begin{array}{cc} \frac{{\left[\tau_{ij}\right]}^{\alpha }{\left[\eta_{ij}\right]}^{\beta }}{\sum_{j\in N_{i}^{K}}{\left[\tau_{ij}\right]}^{\alpha }{\left[\eta_{ij}\right]}^{\beta }}; & j\in N_{i}^{K}\\ 0; & \text{otherwise,} \end{array}\right. \end{array}$$

where *η*_*ij*_ is equal to the intensity of failure propagation *I*_*ij*_^*K*^ from node *i* to node *j*, *τ*_*ij*_ is the amount of pheromone deposited for transition from node *i* to node *j*, *α* ≥ 0  and  *β* ≥ 1 are parameters to control the influence of *τ*_*ij*_ and *η*_*ij*_, respectively, and *N*_*i*_^1^ is the set of nodes which connect with node *i*.

The pheromones are updated by

(12) $$\begin{array}{c} \tau_{ij}\left(\;t+1\;\right)=\left(\;1-\rho \;\right)\tau_{ij}\left(\;t\;\right)+\overset{N_{a}}{\underset{K=1}{\sum }}\Delta \tau_{ij}^{K}, \end{array}$$

where *ρ* represents the pheromone evaporation coefficient; *τ*_*ij*_(0) is the initialization of pheromone; ∑_*K*=1_^*N*_*a*_^Δ*τ*_*ij*_^*K*^ is the amount of pheromone; *N*_*a*_ is the number of ants; and Δ*τ*_*ij*_^*K*^ is the pheromone of ant *K*.

### 2.3. Failure Sample Selection Optimization Based on Subsequent Propagation Failure Set

In testability demonstration experiment, for Unit Under Test (UUT), there is a replaceable unit *U*_*i*_  (1 ≤ *i* ≤ *K*,  *K* being the amount of replaceable units), which consists of *N*_*i*_ failure modes. Adopting the rule of allocation in stratified sampling, *m*_*i*_ failure modes are assigned to the replaceable unit  *U*_*i*_. Thus, we need to consider selecting *m*_*i*_ suitable failure modes from total *N*_*i*_ failure modes to establish a failure sample set. As our discussion above, we also need to take the influence of the failure propagation into account. Here, subsequent failure propagation sets (SFPS) are made use of to optimize the failure sample set. SFPS is defined as a set of failure modes which occur in a failure propagation path and it indicates the range of failure spread.

We assume that failure mode set of the replaceable unit *U*_*i*_ is *F*_*i*_ = {*f*_1_, *f*_2_, *f*_3_,…, *f*_*N*_*i*__}, and steps of failure sample selection optimization based on SFPS are described as follows.

**Step 1 .**

Count the SFPS number of every element (failure mode) of the failure mode set *F*_*i*_ to construct a set *I*_*i*_ = {*I*_1_, *I*_2_, *I*_3_,…, *I*_*N*_*i*__}. Then count the number of elements which are greater than 1 in the set *I*_*i*_, marked as *K*_*i*_. After that, select *K*_*i*_ failure modes from *F*_*i*_ to construct a new failure mode set *FI*_*i*_ = {*FI*_1_, *FI*_2_, *FI*_3_,…, *FI*_*K*_*i*__}. These selected failure modes have more than one SFPS. Next, make *F*_*j*_ = *F*_*i*_ − *FI*_*i*_, where *F*_*j*_ is the set of the remaining failure modes.

**Step 2 .**

If *K*_*i*_ ≤ *m*_*i*_, generate a (*m*_*i*_ − *K*_*i*_) × 1 random number set in which these numbers are discrete uniform distribution between 1 and *N*_*i*_ − *K*_*i*_. It is denoted by *SN*_*i*_ = {*S*_1_, *S*_2_, *S*_3_,…, *S*_*m*_*i*_−*K*_*i*__}. And then, according to *SN*_*i*_, extract *S*_1_, *S*_2_,…, *S*_*m*_*i*_−*k*_*i*__ failure modes, respectively, from the set *F*_*j*_ by natural order to make up a set *FS*_*i*_ = {*F*S_1_, *FS*_2_, *FS*_3_,…, *FS*_*m*_*i*_−*K*_*i*__}. A last failure sample set for test ability demonstration experiment is confirmed by *FF*_*i*_ = *FI*_*i*_ ∪ *FS*_*i*_.

**Step 3 .**

If *K*_*i*_ > *m*_*i*_, create *m*_*i*_ random numbers with discrete uniform distribution between 1 and *m*_*i*_. It is marked *SM*_*i*_ = {*M*_1_, *M*_2_, *M*_3_,…, *M*_*m*_*i*__}. Next, select *M*_1_, *M*_2_,…, *M*_*m*_*i*__ elements from the set *FI*_*i*_ to compose a set *FS*_*i*_; *FS*_*i*_ = {*FS*_1_, *FS*_2_, *FS*_3_,…, *FS*_*m*_*i*__} = {*FI*_1_, *FI*_2_, *FI*_3_,…, *FI*_*m*_*i*__}. It is also the last failure sample set *FF*_*i*_.

**Step 4 .**

Achieve the amount of failure sample set through adding up the failure sample sets from Steps 2 and 3.

## 3. Case Study

A certain type of air-to-air missile system consists of six modules, namely, refrigeration module, vibration control device, rectifier, shear stents, lock system, and the box of circuit. Here, we only take the refrigeration module as an example.  Table 2 shows failure modes of the refrigeration module.

Suppose that we have known these values of *q*_1_, *q*_0_, *α*, and  *β* according to the agreed contract between suppliers and customers. By making use of formula (3) the failure sampling plan (50, 6) is confirmed. It means that 50 failure samples will be assigned to 6 modules with proportional stratified sampling method. We have known the assignment weight *W*_1_ of the refrigeration module in the system is 0.121; thus the number of failure samples assigned to it is 6 based on expression (4). Therefore, we should pick up 6 suitable failure samples from a total of 12 failure samples to establish failure sample set for refrigeration module.

**Figure 4:**
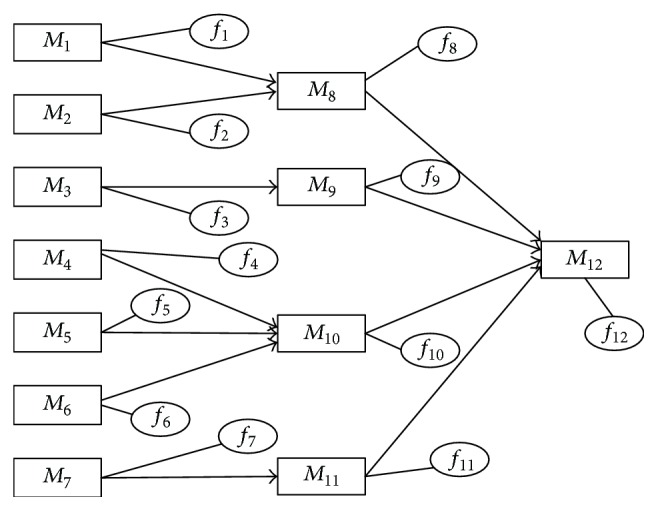
Directional graph of failure probability propagation for refrigeration module.

In accordance with the circuit connection of the system, directional graph of failure propagation for refrigeration module is gained as shown in Figure 4. From the graph, we can see that the component set of refrigeration module is *M* = {*M*_1_, *M*_2_, *M*_3_,…, *M*_12_} and its failure mode set is *F* = {*f*_1_, *f*_2_, *f*_3_,…, *f*_12_}. To take advantage of formulas (6), (7), and (8), the propagation intensity of each directed edge in the system is obtained as shown in Table 3. In the table, the range of *Z*-score value is from −1.1389 to 1.6663.

Analyzing the failure propagation with the data from Table 3, it is clear that the greater the *Z*-score value of an edge is, the more possible it is that the failure propagation happens on the edge. For instance, the *Z*-score value of intensity of edge (*M*_7_, *M*_11_) is −1.1389. It is the smallest value in all *Z*-score values. This means that when a failure occurs in *M*_7_, the failure could not spread to *M*_11_ or spread to *M*_11_ with tiny possibility. However, edge (*M*_3_, *M*_9_) has the greatest *Z*-score value of 1.663. It shows propagation failure is inevitable on the edge. Thus, through searching the maximum *Z*-score for each failure with ACO, we can find the maximum probability propagation path of failure easily. Take *M*_1_ as an example; failure *f*_1_ spreads along with edge (*M*_1_, *M*_8_) and edge (*M*_8_, *M*_12_) which have the biggest intensities (0.8528 and 1.6663, resp.). As a result, the maximum probability propagation path (*M*_1_ → *M*_8_ → *M*_12_) of failure *f*_1_ is obtained with ACO. At the next step, we use the method mentioned in Section 2.3 to obtain the related subsequent failure propagation set *F*_PossibilitySet_ (*f*_1_) of *f*_1_; *F*_PossibilitySet_  (*f*_1_) = {*f*_8_, *f*_12_}. It means the failure spread number from *M*_1_ is 2. Utilizing the same method, other modules' subsequent failure propagation sets and failure spread numbers can be solved as well. Finally, the optimization samples set is established based on the failure spread path.

Table 4 shows the advantages of the proposed method compared with traditional failure sampling plan. The symbol √ in the table expresses the selection of failure samples. By the traditional sample plan, 6 samples are assigned randomly to 12 modules, not taking into account the influence of failure propagation. Conversely, the proposed method can reasonably choose 6 samples under the consideration for failure propagation. Through experiment, the proposed method has better failure coverage than traditional one.

## 4. Conclusion

This paper proposes a new failure sample selection method to cover the shortage of the traditional sample selection. First of all, we use the DG and ACO to obtain a maximum probability failure propagation path based on the intensity of edge. Then we proposed the new failure sample selection method on the basis of the subsequent failure propagation set. Compared with traditional sampling plan, this method is able to increase the coverage of failure due to establishing a relatively complete fault sample set through focusing on the propagation failure and a case study is given to demonstrate that it can decrease the risk of using.

## Figures and Tables

**Table 1:** The failure propagation intensity of each edge.

	Directed edge			
	(*M*_1_, *M*_3_)	(*M*_2_, *M*_3_)	(*M*_2_, *M*_4_)	(*M*_4_, *M*_5_)
Intensity of edge	0.2	0.1	0.4	0.2
*Z*-score value	−0.1987	−0.9934	1.3908	−0.1987

**Table 2:** Failure modes of the refrigeration module.

Components	Fault	Failure mode
*M*_1_	*f*_1_	Seal A failure
*M*_2_	*f*_2_	Check valve failure
*M*_3_	*f*_3_	Pipeline failure
*M*_4_	*f*_4_	Exhaust port failure
*M*_5_	*f*_5_	Seal B failure
*M*_6_	*f*_6_	Adapter defect
*M*_7_	*f*_7_	Electromagnetic switch failure
*M*_8_	*f*_8_	Charging valve leakage
*M*_9_	*f*_9_	Refrigeration pipe deformation
*M*_10_	*f*_10_	Gauges failure
*M*_11_	*f*_11_	Electromagnetic switch leakage
*M*_12_	*f*_12_	Refrigeration elements failure

**Table 3:** The failure propagation intensity of each edge in refrigeration module.

Directed edge	Propagation intensity	*Z*-score value
(*M*_1_, *M*_8_)	*I*_1,8_ = 0.710	0.8528
(*M*_2_, *M*_8_)	*I*_2,8_ = 0.290	−0.3254
(*M*_3_, *M*_9_)	*I*_3,9_ = 1.000	1.6663
(*M*_4_, *M*_10_)	*I*_4,10_ = 0.500	0.2637
(*M*_5_, *M*_10_)	*I*_5,10_ = 0.250	−0.4376
(*M*_10_, *M*_12_)	*I*_10,12_ = 0.000	−1.1389
(*M*_6_, *M*_10_)	*I*_6,10_ = 0.167	−0.6704
(*M*_7_, *M*_11_)	*I*_7,11_ = 0.000	−1.1389
(*M*_8_, *M*_12_)	*I*_8,12_ = 1.000	1.6663
(*M*_9_, *M*_12_)	*I*_9,12_ = 0.226	−0.5049
(*M*_11_, *M*_12_)	*I*_11,12_ = 0.323	−0.2328

**Table 4:** Comparison of traditional sampling plan and the proposed method.

Name	Failure modes	SFPS number	Traditional	Proposed
*M*_1_	Seal A failure	2	√	√
*M*_2_	Check valve failure	2	√	
*M*_3_	Pipeline failure	2	√	√
*M*_4_	Exhaust port failure	1		
*M*_5_	Seal B failure	0		√
*M*_6_	Adapter defect	2	√	√
*M*_7_	Electromagnetic switch failure	0		
*M*_8_	Charging valve leakage	1	√	√
*M*_9_	Refrigeration pipe deformation	0	√	
*M*_10_	Gauges failure	1		
*M*_11_	Electromagnetic switch leakage	1		√
*M*_12_	Refrigeration elements failure	1		
